# Reinforcement sensitivity and restrained eating: the moderating role of executive control

**DOI:** 10.1007/s40519-016-0343-z

**Published:** 2016-11-25

**Authors:** Nienke C. Jonker, Elise C. Bennik, Peter J. de Jong

**Affiliations:** Department of Clinical Psychology and Experimental Psychopathology, University of Groningen, Grote Kruisstraat 2/1, 9712 TS Groningen, The Netherlands

**Keywords:** Restrained eating, Reinforcement sensitivity, Reward sensitivity, Reward responsivity, Punishment sensitivity, Executive control

## Abstract

**Purpose:**

As the prevalence of overweight and obesity are still increasing, it is important to help individuals who encounter difficulty with losing weight. The current study was set out to further investigate characteristics of individuals who are highly motivated to restrict their food intake to lose weight, but fail to do so (i.e., restrained eaters). The motivation to lose weight might stem from high punishment sensitivity, whereas the failure to succeed in restricting food intake might be the result of high reward sensitivity. Thus, it was examined whether restrained eaters are characterized by both high reward sensitivity and high punishment sensitivity. Additionally, this is the first study to examine executive control as a potential moderator of this relationship.

**Methods:**

Female undergraduates (*N* = 60) performed a behavioral measure of executive control, and completed the Restraint Scale to index level of restrained eating as well as two questionnaires on reinforcement sensitivity; the Behavioral Inhibition Scale/Behavioral Activation Scale, and the Sensitivity to Punishment and Sensitivity to Reward Questionnaire.

**Results:**

There was a positive relationship between restrained eating and punishment sensitivity as indexed by both questionnaires. Reward sensitivity as measured by both indices was not directly related to restrained eating. Executive control moderated the relation between reward responsivity (but not reward-drive) and restrained eating; specifically in women with relatively weak executive control there was a positive relationship between reward responsivity and restrained eating behavior.

**Conclusion:**

In women with low executive control, restrained eating is associated with both heightened sensitivity to punishment and heightened responsivity to reward.

## Introduction

Dieting has become a normal part of society, especially for women. Underlining its common nature, a large scale cross-cultural study found that 51% of young adolescent female participants indicated to be currently trying to lose weight (*N* = 18.512, from 22 countries) [1]. However, since the prevalence of overweight and obesity in women are increasing (from 29.8% in 1980 to 38.0% in 2013) [2], it is important to help individuals who encounter difficulty with losing weight. An important step is to identify characteristics of individuals who are highly motivated to restrict their food intake to lose weight, but fail to do so (i.e., restrained eaters) [3–5]. Characteristics that may be related to restrained eating are reward and punishment sensitivity.

Individuals with an enhanced sensitivity to rewards are more inclined to respond with approach behavior in situations that are related to reward [6, 7]. Individuals who are sensitive to punishment are more prone to respond with avoidance behavior in situations that are related to punishment [6, 7]. On top of the relation with normal behavior, reward and punishment sensitivity have also been found to relate to symptoms of psychopathology such as substance misuse, anxiety, and eating disorders [8].

As opposed to restricted eaters, such as Anorexia Nervosa patients, who have been mainly found to be characterized by high punishment sensitivity [9], restrained eaters might be characterized by both high reward sensitivity and high punishment sensitivity. Individuals with high reward sensitivity are thought to be relatively sensitive to the rewarding features of eating high caloric food items, which may lower the threshold for overeating [10]. Further, individuals with high punishment sensitivity are thought to be more likely to restrict their food intake, since they are more inclined to avoid the punishing consequences of overeating such as becoming overweight and obese [11]. Hence, restrained eaters might be characterized by both high reward sensitivity and high punishment sensitivity. The motivation to lose weight might stem from their punishment sensitivity, whereas the failure to succeed in restricting food intake might be the result of high reward sensitivity.

However, previous studies have shown inconsistent results with regard to the relationship between reward and punishment sensitivity and restrained eating. Female adolescent restrained eaters have indeed reported a higher sensitivity to reward and a higher sensitivity to punishment on the Sensitivity to Punishment and Sensitivity to Reward Questionnaire (SPSRQ) [12], than unrestrained eaters [13]. Though, another study among young adolescents found that only reward sensitivity was positively related to restrained eating in girls, whereas punishment sensitivity was only positively related to restrained eating in boys [14]. In this study, reward and punishment sensitivity were measured with the Behavioral Inhibition Scale/Behavioral Activation Scale (BIS/BAS) [15]. In a study among adults (combined male/female sample) in which the BIS/BAS was used, reward sensitivity, but not punishment sensitivity, was found to be positively related to restrained eating [16]. At last, in a study using both the BIS/BAS and the SPSRQ among young adolescents (combined male/female sample), punishment sensitivity measured with both indices was found to be positively related to restrained eating, whereas reward sensitivity measured with both questionnaires was not [17].

A possible explanation for these inconsistent findings is the difference in measures that were used to index reward and punishment sensitivity. Although the SPSRQ and the BIS/BAS questionnaires have been used interchangeably as indices for reinforcement sensitivity, they are not identical. The SPSRQ seems to be a more context and stimulus dependent measure than the BIS/BAS [11]. The choice of a particular scale appears to especially impact the results with respect to the relationship between eating problems and reward sensitivity. For example, studies using the BIS/BAS have consistently found that patients with anorexia nervosa report lower reward sensitivity than healthy controls [18, 19], whereas patients with anorexia were found to report higher reward sensitivity than healthy controls when using the SPSRQ [11, 19]. Importantly, the difference between patients and healthy controls completely disappeared after excluding the items of the SPSRQ that refer to appearance and interpersonal reward elicitors (e.g., “Do you often meet people that you find physically attractive?”) [11].

Additionally, the inconsistent findings might be the result of a third factor influencing the relation between reinforcement sensitivity and restrained eating. Theoretical models on addiction and related behavior (e.g., dysregulated eating behaviors) emphasize the importance of both bottom-up motivational processes (e.g., automatically triggered approach responses towards food cues), and top-down control processes, fueled by long-term considerations that contradict the pursuit of unhealthy incentives [20]. Accordingly, the failure of restrained eaters to comply with their diet goal may not only be driven by relatively high sensitivity for the rewarding properties of food items (i.e., bottom-up processes), but also by a deficient top-down regulation of their automatic approach behavior. Self-regulation in terms of resisting the temptation of food requires adequate executive control (EC) [21]. Therefore, people with low EC may lack the ability to successfully redirect their thoughts and actions towards obtaining their diet goal. Thus, especially individuals with both high reward sensitivity and weak EC may be unable to resist (food) rewards, and will therefore experience problems with restricting their food intake. In other words, EC might moderate the relationship between reward sensitivity and restrained eating. Since high punishment sensitivity is thought to be in line with restrained eaters’ goal of losing weight and restricting their food intake, there is no reason to assume that the relationship between punishment sensitivity and restrained eating will be moderated by EC.

All in all, the first aim of the current study is to further examine the relationship between restrained eating and reinforcement sensitivity. To investigate whether inconsistencies in previous research might have been due to the indexes that were used to measure reinforcement sensitivity, we will include both the BIS/BAS and the SPSRQ. To get more fine-grained insight in the components of reward sensitivity that are related to restrained eating, the focus is not only on the complete reward scale of the BIS/BAS, but follow-up analyses will be done with regard to the reward responsivity and reward drive subscales [6, 7]. With regard to the SPSRQ, we will not only examine the relationship between the original reward sensitivity subscale and restrained eating, but also between the reward scale after excluding the items referring to appearance and interpersonal rewards as proposed by Glashouwer and colleagues [11]. The second aim of the current study is to examine whether EC moderates the relationship between reinforcement sensitivity and restrained eating.

## Method

### Participants

The final samples of 60 female undergraduate students of the University of Groningen were selected during the first two months of the academic year from a group of 152 students, who were on-line screened with the Restraint Scale (RS) [21], for being either relatively low (score <9) or relatively high restrained eaters (score >16). Only women were selected, since they are more likely to show restrained eating and dieting behavior than men [1]. During the subsequent laboratory assessments, the selected participants again completed the RS. Although originally selected on the basis of extreme scores, results showed that there was considerable regression to the mean, resulting in an approximately normal distribution of RS scores. Following the recommendation of Preacher and colleagues [22], a correlational approach was preferred over an extreme groups approach (see Table 1 for sample characteristics).

**Table 1:** Sample characteristics (*N* = 60)

	Mean	SD
Age	19.73	1.45
Restrained eating	13.38	6.97
BMI	21.65	3.84

## Materials

### Restrained eating behavior

Restrained eating behavior was indexed by the Restraint Scale [23], aimed to identify unsuccessful dieters with a tendency to overeat [5]. The scale consists of 10 items that are answered on a 4-point or 5-point scale, and total scores can range from 0 to 35 (e.g., “Do you eat sensibly in front of others and splurge alone”). Internal reliability of the restraint score in the current study was good (Cronbach’s alpha = 0.87).

### Reward and punishment sensitivity

Self-reported reward and punishment sensitivity were measured with two different questionnaires, the BIS/BAS and the SPSRQ.

*BIS/BAS* The BIS/BAS [15] contains 24 items including 4 distracter items. Items are answered on a 4-point scale ranging from; (1) very false for me, to (4) very true for me. The questionnaire consists of two main subscales; punishment sensitivity (BIS; e.g., “Criticism or scolding hurts me quite a bit”), and reward sensitivity (BAS-total). The reward sensitivity scale can be split into three subscales; reward responsiveness (BAS-RR; e.g., “When I am doing well at something, I love to keep at it”), reward drive (BAS-Drive; e.g., “I go out of my way to get things I want”), and fun seeking. Scores on the subscales were computed by averaging the item scores. Reliability values of the BIS, BAS-total, BAS-RR, and BAS-Drive scales were good to acceptable (Cronbach’s alpha of 0.70, 0.78, 0.79, and 0.60, respectively). The fun seeking subscale was not of interest in the current study, yet also had poor reliability in terms of internal consistency (Cronbach’s alpha of 0.35).

*SPSRQ* The SPSRQ [12] contains 24 questions about sensitivity to reward (SR; e.g., “Do you often do things to be praised?”), and 24 questions about sensitivity to punishment (SP; e.g., “Are you easily discouraged in difficult situations?”). These questions are answered with yes or no. Scores on both subscales represent the sum of the items that were answered with yes. Reliability of the SR and SP subscales in the current study was good (Cronbach’s alpha of 0.77, and 0.83, respectively). A second reward sensitivity subscale score was calculated based on Glashouwer et al. [11], excluding the items regarding appearance and interpersonal rewards (e.g., “Do you often meet people that you find physically attractive?”). This subscale showed good reliability (Cronbach’s alpha of 0.74).

### Executive control (EC)

The Attentional Network Task (ANT) was used as a behavioral measure of EC [24]. The ANT is a computer task during which participants have to determine whether an arrow on the screen points to the left or right. This arrow is accompanied by flankers that are either congruent or incongruent. In between trials, participants have to fixate their attention on a point that is shown in the middle of the screen. The arrows appear either above or below this central fixation point. There are trials in which a cue is given just before the arrows appear (center cue), trials in which the cue signals where the target is coming (up or down; spatial cue), and trials without a cue. The task starts with 24 practice trials, followed by 144 experimental trials. During the experimental trials, all combinations of flanker type (congruent, incongruent), cue type (center cue, spatial cue, no cue), and position (up or down) were presented six times. EC scores were calculated by subtracting the mean reaction time (RT) on congruent trials from the mean RT on incongruent trials. A lower score reflects better EC.

### Procedure

The current study was part of a two-session study approved by the ethical committee of the psychology department of the University of Groningen. Participants were invited to the lab via email, and received study credits for their participation. After receiving information about the study they signed the informed consent. The first session took approximately 45 min, and started with two computer tasks, among which the Attentional Network Task. The ANT was programmed in E-prime 2.0 (Psychology Software Tools, Pittsburgh, PA, USA), and was run on a Windows 7 computer with a 27 inch LED screen (1920 × 1080, 60 Hz). After the ANT, participants filled out the BIS/BAS first, followed by the SPSRQ. To disguise that the study was about eating behavior, the restraint scale was administered at the end of the second session, which was administered between 6 and 28 days after the first session. This second session was administered for an unrelated study, only the restraint scale was used from this second session.

### Analyses

After checking the relevant assumptions, hierarchical regression analyses were performed with restrained eating as dependent variable. In the first step, reward and punishment sensitivity were entered, EC was entered in the second step, and in the third step the interaction between reward sensitivity and EC, and punishment sensitivity and EC were entered. Analyses were performed separately for reward and punishment sensitivity scores of the BIS/BAS (A) and the SPSRQ (B). The main BIS/BAS analysis was followed by two regression models in which the BAS-Drive (A2), and the BAS-RR (A3) subscales were entered instead of the BAS-total scale (A1). The main SPSRQ analysis was followed by a regression model in which SR excluding items regarding appearance and interpersonal rewards was entered (B2) instead of the total SR scale (B1). Since we tested our hypotheses with two different questionnaires we used a corrected alpha of 0.025 (α of 0.05/2).

## Results

### Data reduction and descriptive statistics

Before EC scores were calculated from the attentional network task, RTs of trials with incorrect responses (2.8%) and outliers (>2.5 SD from the mean; 2.3%) were removed. Subsequently, the mean RTs on congruent trials were subtracted from the mean RTs on incongruent trials resulting in an EC score. Descriptive statistics are shown in Table 2.

**Table 2:** Descriptive statistics (*N* = 60)

	Mean	SD
BAS-Total	2.00	0.36
BAS-Rr	1.64	0.38
BAS-Dr	2.41	0.59
BIS	2.11	0.32
SR	11.30	4.15
SR-2	7.87	3.10
SP	11.18	4.91
Executive control	77.31	24.79

*BAS-Total* BIS/BAS Reward total, *BAS-Rr* BIS/BAS Reward responsiveness, *BAS-Dr* BIS/BAS Reward drive, *BIS* BIS/BAS punishment sensitivity, *SR* SPSRQ reward sensitivity, *SR-2* SPSRQ reward sensitivity adapted following Glashouwer et al. [11], *SP* SPSRQ punishment sensitivity

Bivariate correlational analysis (see Table 3) showed that restrained eating was positively related to BMI and punishment sensitivity as measured with the BIS/BAS. Yet, there were no significant correlations between restrained eating and punishment sensitivity as measured with the SPSRQ, reward sensitivity as measured with both questionnaires, or EC.

**Table 3:** Bivariate correlations between restrained eating, BMI, reward sensitivity, punishment sensitivity, and executive control measures

	Restrained eating	BMI^a^	BAS-Total	BAS-Rr	BAS-Dr	BIS	SR	SR_2	SP
BMI^a^	0.52***	–	–	–	–	–	–	–	–
BAS-T	−0.06	−0.15	–	–	–	–	–	–	–
BAS-Rr	0.00	−0.12	0.78***	–	–	–	–	–	–
BAS-Dr	−0.08	−0.13	0.86***	0.49***	–	–	–	–	–
BIS	0.37**	0.16	−0.02	0.20	−0.10	–	–	–	–
SR	0.14	−0.01	0.56***	0.41**	0.46***	0.09	–	–	–
SR_2	0.17	−0.02	0.61***	0.38**	0.52***	0.03	0.92***	–	–
SP	0.23	0.25	−0.34**	−0.17	−0.33*	0.56***	−0.17	−0.28*	–
EC	0.02	−0.19	−0.09	0.05	−0.23	0.02	0.06	0.06	0.08

*BAS-T* BIS/BAS reward total, *BAS-Rr* BIS/BAS reward responsiveness, *BAS-Dr* BIS/BAS reward drive, *BIS* BIS/BAS punishment sensitivity, *SR* SPSRQ reward sensitivity, *SR_2* SPSRQ reward sensitivity adapted following Glashouwer et al. [11], *SP* SPSRQ punishment sensitivity, *EC* Executive control

* *p* < 0.05

** *p* < 0.01

*** *p* < 0.001

^a^Spearmans rho correlations, because of violations of normality

### Hierarchical regression analyses

#### Reward sensitivity (BAS), punishment sensitivity (BIS) as measured with the BIS/BAS, executive control (EC), and restrained eating

The hierarchical regression model with BIS and BAS-total (model A1) showed no significant main effects of BAS-total and EC on restrained eating. BIS was a significant predictor of restrained eating. Additionally, the interaction effect between BAS-total and EC was not significant. The interaction between BIS and EC did not significantly predict restrained eating. The analysis was also performed for the BAS-Drive (model A2) and BAS-Reward Responsivity (BAS-RR) (model A3) subscales specifically. The results with regard to BIS remained the same. Additionally, no main effects were found for BAS-RR, and BAS-Drive, or an interaction effect between BAS-drive and EC. Yet, the interaction between BAS-RR and EC was significant (see Table 4).

**Table 4:** Hierarchical regression analysis of BIS/BAS subscales on restrained eating

Model	Variable	*B*	SEB	*T*	*p*	Adj-R2 (%)
A1	BAS	0.50	2.45	0.20	0.839	11
	BIS	5.77	2.00	2.89	0.006	
	EC	0.03	0.04	0.80	0.429	
	BAS × EC	0.21	0.11	1.87	0.067	
	BIS × EC	0.02	0.09	0.17	0.870	
A2	BAS-Dr	1.26	1.61	0.78	0.438	7
	BIS	6.10	2.01	2.97	0.004	
	EC	0.02	0.04	0.45	0.658	
	BAS-Dr × EC	0.02	0.05	0.36	0.722	
	BIS × EC	0.00	0.10	0.03	0.973	
A3	BAS-RR	−0.24	2.34	0.10	0.920	15
	BIS	4.73	2.01	2.27	0.027	
	EC	0.02	0.04	0.54	0.594	
	BAS-RR × EC	0.33	0.13	2.44	0.018	
	BIS × EC	−0.06	0.10	−0.58	0.566	
A4	BAS-RR	−0.24	2.34	0.10	0.836	15
	BIS	4.73	2.01	2.27	0.049	
	EC	0.02	0.04	0.54	0.120	
	BMI	1.12	0.28	4.02	<0.001	
	BAS-RR × EC	0.33	0.13	2.44	0.030	
	BIS × EC	−0.06	0.10	−0.58	0.259	
	BMIxEC	0.03	0.01	2.73	0.009	

*BAS-Total* BIS/BAS reward total, *BAS-Rr* BIS/BAS reward responsiveness, *BAS-Dr* BIS/BAS reward drive, *BIS* BIS/BAS punishment sensitivity, *EC* executive control

**Fig. 1:**
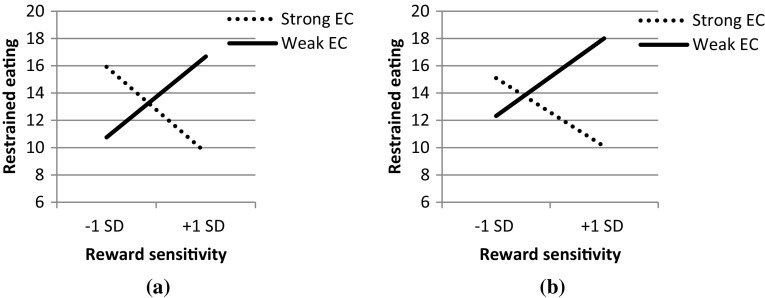
Interaction effect of reward responsivity and executive control on restrained eating, **a** original model, **b** after controlling for BMI and BMIxEC

To further investigate the significant interaction between BAS-RR and EC on restrained eating, simple slopes were plotted for strong (−1 SD below the mean) and weak (+1 SD above the mean) levels of EC (See Fig. 1a). A positive relation was found between reward responsivity and restrained eating for individuals with weak EC, and a negative relation between reward responsivity and restrained eating for individuals with strong EC. Since there was a relation between both EC and BMI (*r* = −0.19), and reward responsivity and BMI (*r* = −0.12), a post hoc analysis was performed to examine whether the interaction between EC and BMI influences the interaction between reward responsivity and EC on restrained eating (model A4; Table 4). After controlling for BMI and ECxBMI, it was found that individuals with a high reward responsivity and low EC were most inclined to show restrained eating, whereas individuals with high reward responsivity and high EC were least inclined to (Fig. 1b).

#### Reward sensitivity (SR) and punishment sensitivity (PS) as measured with the SPSRQ, executive control (EC), and restrained eating

The hierarchical regression model with SP and SR (model B1 and B2) showed that SR (with and without excluding items regarding appearance and interpersonal rewards), and the interaction between SR and EC did not significantly predict restrained eating. In the model including the original reward sensitivity, SP, and the interaction between SP and EC were not significant predictors of restrained eating. After excluding the items regarding appearance and interpersonal rewards of the reward sensitivity subscale (model B2), only SP was a marginally significant predictor of restrained eating behavior. The interaction between SP and EC was not a significant predictor of restrained eating (See Table 5).

**Table 5:** Hierarchical regression analysis of SPSRQ subscales on restrained eating

Model	Variable	*B*	SEB	*T*	*p*	Adj-R2
B1	SR	0.32	0.02	1.49	0.142	7%
	SP	0.35	0.18	1.94	0.057	
	EC	0.01	0.04	0.32	0.750	
	SR × EC	0.01	0.01	0.62	0.535	
	SP × EC	−0.02	0.01	−1.93	0.059	
B2	SR-2	0.50	0.29	1.72	0.091	10%
	SP	0.42	0.18	2.28	0.026	
	EC	0.01	0.04	0.39	0.696	
	SR-2 × EC	0.02	0.01	1.22	0.227	
	SP × EC	−0.02	0.01	−1.67	0.102	

*SR* SPSRQ reward sensitivity, *SR-2* SPSRQ reward sensitivity adapted following Glashouwer et al. [11], *SP* SPSRQ punishment sensitivity, *EC* executive control

## Discussion

The current study was set out to further investigate characteristics of individuals who are highly motivated to restrict their food intake but fail to do so, as an important step in the search for better help for individuals who encounter difficulty with losing weight. The major findings of the current study can be summarized as follows: (1) restrained eating was positively related to punishment sensitivity as indexed by both the BIS/BAS and the SPSRQ; (2) reward sensitivity as indexed by both indices was not directly related to restrained eating; and (3) EC moderated the relationship between restrained eating and reward responsivity as indexed by the BAS; only for those with relatively low EC there was a positive relationship between reward responsivity and restrained eating.

In line with our hypothesis, restrained eating was associated with relatively high punishment sensitivity. Underlining the robustness of the association between restrained eating and punishment sensitivity, this positive relationship was evident for both indices. Thus, women who indicated to be more sensitive to cues of punishment were more inclined to engage in restrained eating behavior. This is consistent with the idea that punishment sensitivity is related to the motivation to diet. In line with this, previous findings show that anorexia nervosa patients are also characterized by relatively high punishment sensitivity [11]. The relationship between punishment sensitivity and restrained eating that was evident in the current study is consistent with some previous findings [13, 17], yet, not with all [14, 16]. These inconsistencies could not be explained by the use of a particular punishment sensitivity measure, since the relation was found for both the BIS/BAS and the SPSRQ. Yet, differences in sample characteristics might explain the inconsistent findings. Average levels of punishment sensitivity have been found to differ between age groups and sex [25], and there seem to be sex differences in the relationship between punishment sensitivity and restrained eating [14]. The sample of Ahern et al. [13] closely resembles our sample. Participants in the study by Stapleton and Whitehead [16] were much older, and consisted of both males and females, whereas participants in the study by Walther and Hilbert [14] were much younger. Thus, it seems that punishment sensitivity might be mainly related to restrained eating during adolescence and young adulthood.

The current findings did not support the view that heightened reward sensitivity has a direct relationship with restrained eating. Thus, the results do not support the hypothesis that the failure to succeed in restricting food intake is directly related to high reward sensitivity. This could not be attributed to the use of a particular measure of reward sensitivity as none of the indices of reward sensitivity showed a meaningful bivariate association with restrained eating behavior. This is consistent with a study among young adolescents that also failed to find a relationship between restrained eating and reward sensitivity as indexed by either the BIS/BAS or the SPSRQ [17]. The current findings challenge the robustness of earlier results suggesting a direct positive relationship between reward sensitivity and restrained eating [13, 14, 16]. One explanation for the divergence of results concerns differences in measures that were used to index-restrained eating behavior. The studies that did find a direct relationship between reward sensitivity and restrained eating used the Dutch Eating Behavior Questionnaire (DEBQ) [26] to index-restrained eating. It has been suggested that the DEBQ more closely reflects successful dieting, whereas the restraint scale seems to identify unsuccessful dieters with a tendency to overeat [5]. Perhaps, then, the relationship between reward sensitivity and successful dieting (DEBQ-restraint) is more closely linked to the long-term reward of favorable effects on weight than by the immediate reward of eating food items.

The current study shows that it is important to examine individual differences in EC when examining the relation between reinforcement sensitivity and restrained eating. Individuals with a strong EC are expected to show an enhanced ability to inhibit automatic responses that are inconsistent with their current goals. Therefore, it was anticipated that the relationship between reward sensitivity and restrained eating behavior would be especially evident in women with relatively weak EC. In line with this, results showed that the relationship between reward sensitivity as indexed by the BAS reward responsivity and restrained eating behavior was moderated by EC. Especially for women with a relatively low ability to inhibit automatic responses, there was a positive relationship between reward responsiveness and restrained eating. It would be important for future research to examine whether the relationship between reward sensitivity and other types of eating behavior or eating disorders are similarly moderated by individual differences in EC. For example, reward sensitivity might be especially relevant in the context of Bulimia Nervosa for those with relatively low EC. At the same time, it could be that high reward and punishment sensitivity might be especially linked to restrictive eating disorders such as Anorexia Nervosa in those with relatively high levels of EC, which might help explain their ability to persist in restricting their food intake even when being severely underweight.

The current finding that the relationship between reward sensitivity and restrained eating behavior was only evident in women with relatively weak EC suggests that especially for these women enhancing executive control would be a relevant starting point to help counterforce (automatic) approach tendencies that are inconsistent with their diet goal. It would be interesting for future research to test whether training EC would indeed be effective in neutralizing the relationship between heightened reward responsivity and restrained eating [27].

Although the findings of the current study with regard to punishment sensitivity were relatively consistent across measures, the relation between the BIS and restrained eating was stronger than the relation between the SP and restrained eating.^1^ This corroborates the suggestion that the BIS/BAS questionnaire is a more robust and less stimulus-dependent measure than the SPSRQ. Additionally, the current study provided some tentative evidence for the relevance of differentiating between the various dimensions of reward sensitivity as indexed by the BAS. Although none of the indices of reward sensitivity was directly related to restrained eating, specifically reward responsivity was found to interact with EC in relation to restrained eating. In other words, it seems that the relatively positive impact of receiving a reward is related to restrained eating in individuals with weak executive control, and not so much a heightened drive toward getting a reward. Future studies should also consider examining different facets of punishment sensitivity. It might, for example, be that restrained eaters are mainly characterized by an increased need to avoid punishment, and that responsivity to punishment is not relevant. Additionally, it would be important to examine in future research whether also in the context of eating disorders differentiation between the need (to avoid) and responsivity to both reward and punishment is relevant. For example, it might be that mainly reward responsivity is associated with Bulimia Nervosa, whereas in binge eating disorder also the need for reward is relevant.

Some limitations of the study should be taken into account when interpreting the results. First of all, the sample size of the current study was relatively small in relation to the amount of models that were calculated. Future studies should replicate the current study to test the reliability of the findings. Second, participants in the current study were students who are relatively highly educated and therefore expected to have stronger executive control than the average population. The impact of EC might therefore be underestimated by the current study. Hence, it would be important for future research to also examine the relationship between restrained eating, reinforcement sensitivity, and executive control in a less highly educated sample. Additionally, the correlational nature of the study prohibits drawing conclusions about the causality, and the direction of the found relationships. It would therefore be important to complement the current cross-sectional approach with longitudinal studies to test whether heightened reinforcement sensitivity and low EC indeed precede the development of restrained eating. In addition, no information is available regarding other characteristics of the current sample that might have influenced the results such as symptoms of depression and psychosis. It would therefore be important to replicate the current findings in different type of samples, and to more comprehensively assess relevant characteristics that might moderate the relationships between restrained eating, reinforcement sensitivity, and executive control. Furthermore, it has been argued that the restraint scale might be unable to differentiate between successful and unsuccessful dieters [28]. Yet, in the current study and other studies [29], a strong positive correlation was found between restrained eating and BMI thereby supporting the view that the restraint scale is capturing unsuccessful dieters. It might nevertheless be relevant for future studies to include additional measures that may be helpful to differentiate between successful and unsuccessful dieters such as the food-craving questionnaire [30]. At last, a self-report measure of reinforcement sensitivity was used in the current study. Given the automatic nature of reinforcement sensitivity, it is important to replicate the current study with a behavioral measure of reward and punishment sensitivity.

To conclude, we found that heightened punishment sensitivity is related to restrained eating in general, and that heightened reward responsivity (and not reward-drive) is specifically related to restrained eating in individuals with low EC. The current study is the first to show that the relationship between reward responsiveness and restrained eating behavior is moderated by executive control. Additionally, this is the first study to differentiate between different types of reward behavior. Findings show that differentiating between the various dimensions of reward sensitivity when considering the relation with restrained eating is important. A critical next step is to examine whether also the relationship between reinforcement sensitivity and clinical levels of eating behavior problems depends on the level of EC.

## Footnotes

1 Post-hoc performed partial correlational analysis showed that the relation between BIS and restrained eating remained significant after controlling for SP (*r* = 0.29; *p* < 0.03), whereas the relation between SP and restrained eating was not significant after controlling for BIS (*r* = 0.03; *p* = 0.81).
